# Improving primary care identification of familial breast cancer risk using proactive invitation and decision support

**DOI:** 10.1007/s10689-020-00188-z

**Published:** 2020-06-11

**Authors:** Nadeem Qureshi, Brittany Dutton, Stephen Weng, Christina Sheehan, Wendy Chorley, John F. R. Robertson, Denise Kendrick, Joe Kai

**Affiliations:** 1 Division of Primary Care, NIHR School for Primary Care Research, School of Medicine, University of Nottingham, 13th Floor, Tower Building, Nottingham, NG7 2RD UK; 2 University Hospitals Derby & Burton NHS Foundation Trust, Royal Derby Hospital, Derby, UK; 3 School of Medicine, University of Nottingham, Nottingham, UK

**Keywords:** Primary health care, Breast cancer, Family history, Medical genetics, Decision support, Risk assessment

## Abstract

Family history of breast cancer is a key risk factor, accounting for up to 10% of cancers. We evaluated the proactive assessment of familial breast cancer (FBC) risk in primary care. Eligible women (30 to 60 years) were recruited from eight English general practices. Practices were trained on FBC risk assessment. In four randomly-assigned practices, women were invited to complete a validated, postal family history questionnaire, which practice staff inputted into decision support software to determine cancer risk. Those with increased risk were offered specialist referral. Usual care was observed in the other four practices. In intervention practices, 1127/7012 women (16.1%) returned family history questionnaires, comprising 1105 (98%) self-reported white ethnicity and 446 (39.6%) educated to University undergraduate or equivalent qualification, with 119 (10.6%) identified at increased breast cancer risk and offered referral. Sixty-seven (56%) women recommended referral were less than 50 years old. From 66 women attending specialists, 26 (39.4%) were confirmed to have high risk and recommended annual surveillance (40–60 years) and surgical prevention; while 30 (45.5%) were confirmed at moderate risk, with 19 offered annual surveillance (40–50 years). The remaining 10 (15.2%) managed in primary care. None were recommended chemoprevention. In usual care practices, only ten women consulted with concerns about breast cancer family history. This study demonstrated proactive risk assessment in primary care enables accurate identification of women, including many younger women, at increased risk of breast cancer. To improve generalisability across the population, more active methods of engagement need to be explored.

*Trial registration: CRUK Clinical Trials Database* 11779.

**Electronic supplementary material:**

The online version of this article (10.1007/s10689-020-00188-z) contains supplementary material, which is available to authorized users.

## Introduction

Breast cancer is the commonest cancer in women in most countries [1], accounting for 30% of all cancers in women, with a lifetime risk of up to one in eight [2]. Women diagnosed at an early stage have a 5-year survival rate of 92% compared to 13% of women presenting at stage IV [3]. Mammographic screening for early cancer detection and appropriate intervention reduces breast cancer mortality and improves survival [4, 5]. However younger women, not routinely offered screening, may present symptomatically with cancer at a later stage, with greater years of life thus lost [4–6]. This is associated with greater mortality in younger women diagnosed with breast cancer [7]. Further, these younger women are also more likely to have a genetic mutation and contralateral breast cancer [8, 9].

Family history of breast cancer is a key risk factor, with the risk increasing according to the number of relatives [10] and first degree relatives affected, and diagnosis at a younger age [11]. Familial breast cancer accounts for up to 5–10% of diagnosed breast cancer cases, with the most common mutations occurring in the BRCA1/2 genes [12].

Taking a three-generation family history can identify women at increased familial risk for breast cancer [13–15]. English National Institute for Health and Care Excellence (NICE) guidelines recommend those identified at or near population risk (“average” risk) be followed up in primary care, with explanation of their level of risk and advice to update their family history regularly. Based on the nature of their family history, up to 1% of women are estimated to be at high familial risk (lifetime risk ≥ 30%), mainly with hereditary cancer syndromes. Another 2% are at moderate familial risk (17–30% lifetime risk), but do not have a specific cancer syndrome [16]. European Breast Cancer Specialist guidelines have defined high familial risk as greater than 20–30% lifetime risk [17]. More specifically, Dutch guidelines define moderate risk as 20–30% lifetime risk, whilst high risk is over 30% lifetime risk [18].

Identification of familial risk of breast cancer can lead to successful prevention strategies. Prophylactic bilateral mastectomy reduces breast cancer mortality and morbidity in higher familial risk women, particularly those with BRCA1/2 genetic mutations [19, 20]. Further, yearly MRI and/or mammographic surveillance of women at moderate familial risk can reduce breast cancer mortality by up to 40% [21]. This improvement in health outcomes is also identified when offering surveillance to younger women [22, 23]. Chemoprevention can also reduce the risk of developing breast cancer [24, 25]. English NICE guidelines recommend women at high familial risk are offered chemoprevention, and chemoprevention be considered in those at moderate risk [14]. European guidelines do not give specific recommendation on chemoprevention in usual clinical practice [17, 26–28]., Given the evidence for preventive surveillance and intervention, primary care may have a critical role in identifying women’s risk [29].

In UK primary care, standard usual (reactive) practice is to assess women opportunistically when they present with concerns about their family history [14]. There appears to be a similar reactive approach across Europe [30]. However this will fail to identify most women at increased cancer risk, particularly those from less advantaged communities and young women [6]. On the other hand, in busy primary care clinics, familial breast cancer risk assessment may be challenging for family physicians [31], resulting in a significant number of unnecessary referrals to specialists and inappropriate mammographic imaging [26, 32–34]. Family History Risk Assessment Software (FaHRAS) has been helpful for decision support in hospital specialist settings [34]. For the current study, this has been modified, to incorporate information from a family history questionnaire previously validated with patients in primary care [35]. We sought to evaluate the proactive invitation of women for assessment of familial breast cancer risk, with use of decision support, in primary care.

## Methods

This intervention study was performed between May 2014 and September 2015. The study was approved by East Midlands National Research Ethics Service (NRES) Committee (Reference 14/EM/0009). This involved recruiting family practices in central England. Initially, sixteen family practices expressed an interest in the study. After four practices were deemed outside of the catchment areas and another four declined after further information provided, eight practices were recruited to the study. In all eight practices, primary care teams (family physicians, office nurses and administrative staff) received a one-hour education session on the identification of familial breast cancer, based on current national guidelines for England (NICE), including referral pathway for familial cancer risk assessment in hospital specialist care. In the recruitment area, the pathway involved general practitioners referring to the breast cancer service. Prior to the clinic appointment, the patient is sent a family history questionnaire by the service. This questionnaire is reviewed with the familial cancer nurse specialist, who will perform a breast cancer risk assessment. If women in moderate or high-risk categories are interested in chemoprevention, they are offered a further appointment with breast consultant to discuss pros and cons of treatment. Those women at very high risk are referred to regional clinical genetics service to consider genetic testing.

We randomly selected four practices who expressed an interest to adopt this proactive approach, using decision-support tool, and matched them by deprivation (nearest index of multiple deprivation decile [36] to four practices following usual care. In the intervention practices, a familial risk assessment decision-support tool (primary care FaHRAS), using English NICE guidelines automated for risk stratification and referral recommendation, was also presented to staff for use in practice [37]. All women, aged 30 to 60 years, were identified from practices’ electronic medical records. Women with a previously confirmed diagnosis of breast cancer or ovarian cancer, or who had had assessment for familial breast cancer risk in the preceding 12 months were excluded.

Women identified from this search were mailed an invitation and a validated family history questionnaire (FHQ) [35] for completion. The FHQ was returned to their practice to enable primary care assessment of familial risk of breast cancer. No reminder invitation was sent. In addition, aligned to national guideline recommendations and current “usual care”, eligible women who attended the practice with concerns about their family history of breast cancer, over the next 8 months, were invited to complete the questionnaire [14]. Administrative staff in each practice were trained to enter women’s family history information into the familial risk assessment decision-support tool, which generated risk assessment and care advice for their family physician.

Based on current English (NICE) guidelines, there were three possible outcomes following assessment of women’s family history information by the decision support tool; average risk, increased risk or uncertain risk. Table 1 shows further details on familial risk stratification, based on NICE guidelines, and actions for patient following risk assessment.

**Table 1:** Familial Risk Stratification and Management (based on NICE guidelines)

Women’s history	Decision support assessment	Action
No family history	Average risk—“population risk” *(Defined as lifetime risk of breast cancer less than 17%)*	Remained under management of primary care and mailed reassuring familial risk information^b^ and a breast awareness leaflet
Family history of breast cancer but assessed at no increased risk based on national guideline^a^	Average risk—“near population risk” *(Defined as lifetime risk of breast cancer less than 17%)*	Remained under management of primary care and mailed reassuring familial risk information^b^ and a breast awareness leaflet
Family history of breast/ovarian/prostate cancer assessed to be significant based on national guideline^a^	Increased risk *(Women with a significant family history: ≥ 17% lifetime risk of breast cancer)*	Posted a letter from their GP informing them of their increased risk and offered specialist referral. Option to invite patient see physician to discuss prior to referral
Family history of breast/ovarian/prostate of uncertain significance based on national guideline^a^	Uncertain Risk	GPs advised to discuss women’s family history information with a secondary care specialist to determine if they were at *average* or *increased* risk

^a^National Institute for Health and Care Excellence. Familial breast cancer: classification and care of people at risk of familial breast cancer and management of breast cancer and related risks in people with a family history of breast cancer. Update of clinical guideline 14 and 41. (Clinical guideline 164.) 2013. https://guidance.nice.org.uk/CG164

^b^Information document explaining woman at average lifetime risk of breast cancer. Also included information adopated from charity Breakthrough Breast Cancer on what can increase or reduce breast cancer risk

The following measures were assessed: return of completed family history questionnaires (FHQ); accuracy of data entry from questionnaires to primary care FaHRAS tool achieved by primary care administrative staff (evaluated by comparing this with that of research associate, trained by familial cancer nurse specialist, inputting the same family history information into the specialists version of the FaHRAS tool); numbers and proportions of women identified at “increased” risk and at “average” risk, before and after taking account of the “uncertain risk” category; and numbers and proportions of women confirmed to be classified at increased risk following specialist assessment and their subsequent management. Finally, at the end of the intervention period, recruited patients’ hospital and primary care records were reviewed to identify if recommended preventive surveillance or treatment was offered.

In the four other “usual care” practices, women consulting their practice with concerns about breast cancer in their family history, over the same period were provided with care as usual.

Analysis was descriptive, with all outcome measures reported as numbers and percentages. Characteristics of the study population were presented as means and standard deviations for normally distributed continuous variables, medians and interquartile range for non-normally distributed continuous variables, and numbers and percentages for categorical variables. Descriptive analyses were conducted in STATA 15.

## Results

In the four practices using a proactive approach and decision support, 1127 (16.1%) of 7012 eligible women returned completed family history questionnaires for assessment. A further 10 women who consulted in these four proactive practices, with family history concerns, also completed this questionnaire opportunistically. The sociodemographic profile of recruited women is shown in Table 2. Over half of these women (55%) were aged under 50 years. Comparing recruited and non-recruited patients, they had a similar age distribution but ethnicity was better recorded in the recruited patients. Further details on recruited and non-recruited patients is given in Table S1.

**Table 2:** Demographics of Study Participants

	Proactive cohort	
	(N = 1127)	
	N	(%)
Age at time of consent		
30–39	253	22.4
40–49	420	37.3
50–59	391	34.7
60 and above	63	5.6
Ethnicity		
White British or Irish	1105	98
Black (inc. Caribbean, African)	5	0.4
European of Jewish origin	5	0.4
Indian subcontinent	3	0.3
Mediterranean	3	0.3
Other^a^	6	0.6
Education^b^		
High school level	216	19.2
College of equivalent	405	35.9
Higher education	446	39.6
No formal qualifications	48	4.3
Missing	12	1.06
Deprivation^c^		
Most deprived	21	1.9
2	94	8.3
3	65	5.8
4	79	7
5	95	8.4
6	158	14
7	128	11.4
8	150	13.3
9	171	15.2
Least deprived	106	9.4
Missing	61	5.3

^a^Other ethnicities include; White American, New Zealander, Russian, Brazilian, Filipino

^b^High School level is qualification at 16 (GCSEs), College level or equivalent qualification includes vocational, A Level or equivalent NVQ3 qualification, Higher education qualification is University Bachelor degree or equivalent qualification, No formal qualification indicates no qualification on leaving high school

^c^Deprivation analysed using the English Indices of Deprivation, 2007©Crown Copyright 2006

Administrative staff at the practices correctly entered over 99% (1134 of 1137) of patients’ family history data from these questionnaires. For three women, data were incorrectly inputted into software or information on the questionnaire was misinterpreted. In the four usual care practices, 10 women consulted in the same period with concerns about family history of breast cancer.

### Familial risk assessment outcomes

**Fig. 1:**
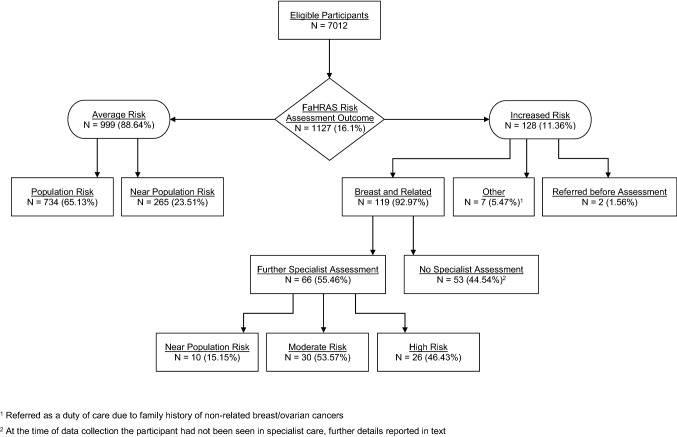
Risk Assessment Outcomes of Women Proactively Invited

Risk assessment outcomes for the 1127 women proactively invited and assessed with decision support are shown in Fig. 1. In total, 999 (88.6%) patients were identified at-or near-population risk (average risk) and managed in primary care, and 128 women (11.4%) were assessed at increased risk with specialist referral recommended, of which 119 offered referral for breast and related cancer risk.

Among the 999 women at average risk, 734 (65.1%) had no family history of breast, ovarian or prostate cancer, and 265 (23.5%) had a family history of such cancers but were assessed as near population risk. 163 of the 265 women were identified by the decision software alone and the remaining 102 women confirmed as near population risk after decision support software recommended discussion with a familial cancer specialist (“uncertain risk” category).

Among the 128 women at increased risk with specialist referral recommended, 64 (50%) women were confirmed by decision software alone, and the remaining 64 (50%) women initially assessed as ‘uncertain’ by decision software were then confirmed to be at increased risk after discussion with a specialist. Of these 128 women at increased risk, 71 women were under 50, and 68 had a family history of breast cancer only, 18 women had a family history of ovarian cancer only, 32 women had a family history of both breast and ovarian cancer, and 1 woman had a family history of prostate cancer. In the remaining 9 women, there were 5 women who had familial colorectal cancer histories, one woman with a cancer syndrome family history, one woman with a sarcoma family history and two women were referred prior to receiving their risk assessment (see Table S2 for full details).

### Outcomes of specialist referral

Outcomes of specialist referral are provided in Table 3. Of 66 women referred during the intervention period, 56 were confirmed to be at increased risk by specialist assessment, 26 (39.4%) were identified at high familial cancer risk of which 24 were offered yearly mammography. A further 30 of these 56 women (45.5%) were classified by a specialist at moderately increased risk with 19 offered mammography. In both high and moderately increased risk categories, confirmatory genetic testing was advised for 15 (26.8%) women’s living relatives (already diagnosed with cancer or with strong family history) rather than genetic testing of the recruited woman. Reviewing the primary and hospital electronic health records indicated none of the high or moderate risk women had been offered chemoprevention during the intervention period. The remaining 10 women were assessed by specialists at near-population risk and returned to be managed in primary care.

**Table 3:** Outcome of referral to familial cancer specialist

Reason	Number	Proportion (%)
**Near-population risk**	**10**	**15.2**
**Moderate increased risk**	**30**	**5.5**
Yearly mammography offered	19	63.3^a^
* Exceeds age for increased surveillance (older than 50)*	10	33.3^a^
* Below the age for increased surveillance (younger than 40)*	6	20^a^
* Breast cancer identified; subsequent bilateral mastectomy*	1	3.33^a^
Additional surveillance		
* Patient referred for genetic mutation testing*	0	0^a^
* Relative referred for genetic mutation testing*	5	13.1^a^
* Genetic test result for relative received*	0	0^a^
**High increased risk**	**26**	**39.4**
Yearly mammography offered	24	92.3^b^
* Exceeds age for increased surveillance (older than 50)*	1	3.85^b^
* Below the age for increased surveillance (younger than 40)*	1	3.85^b^
Additional surveillance		
* Patient referred for genetic mutation testing*	0	0^b^
* Relative referred for genetic mutation testing*	10	38.5^b^
* Genetic test result for relative received*	0	0^b^
**Total**	**66**	

^a^Denominator is based on total patients in moderate risk group

^b^Denominator is based on total patients in high risk group

At the time of data collection in hospital care, 53 of 119 (43.7%) women identified in primary care at increased risk did not have further specialist assessment. This included 16 women referred by their GP who did not attend specialist review (13) or who were referred to another secondary specialist (3); 11 women were not referred because they declined referral (6) or had previously been assessed by familial specialist or genetic services (5); and 21 women who did not respond to their GPs’ further invitations to discuss their results and referral. Finally, 5 women were lost to follow up and no information was available.

### Women assessed opportunistically

Of the further 10 women assessed for familial breast cancer risk in the four “proactive” practices with breast cancer concerns, 3 (all under 50 years old) were identified at increased familial breast cancer risk by decision support with referral recommended. In the other four “usual care” practices, none of the 10 women consulting had a documented family history risk assessment conducted as recommended by national (NICE) guidelines.

## Discussion

This study has found proactive invitation and decision support in primary care enabled the accurate identification of women, including many younger women, at significantly increased risk of breast cancer. Following a single invitation, 16% of eligible women provided their family history information and over 10% of them were newly identified, by decision support in primary care, at increased familial breast cancer risk with specialist referral recommended. The study also demonstrates primary care administrative staff can be trained to enter family history questionnaire, with over 99% of family history data correctly entered into the decision support software. The majority of women referred were confirmed at high or at moderate familial cancer risk by a familial cancer specialist, enabling the initiation of regular mammographic surveillance. One younger woman, aged 46, was thus diagnosed with early breast cancer within the study period itself.

Previous application of decision support in primary and secondary care have improved family history recording and reduced unnecessary referrals [33, 34, 38]. In this study 2.7% of recruited women were classified at moderate familial risk, whist 2.3% at high familial risk. The prevalence of moderate risk women was lower in a Dutch survey (1%) but higher in a Californian survey (6.7%) [39, 40]. The prevalence for women at higher familial risk was lower in this study compared to both the Dutch and Californian surveys. However, both surveys used different definitions for familial risk classification. In the current study, around a third of women at increased risk did not go for further specialist assessment when referred or when offered the opportunity. Continued primary care contact with these women offers the opportunity to heighten awareness of risk and encourage follow up with specialist assessment, particularly if symptoms develop later. Primary care may be best placed to use decision support to inform appropriate future care of these women, including encouraging follow up where active specialist assessment or care is merited or for consideration of preventive treatment.

Women’s engagement following a single postal invitation was relatively modest but the intervention itself reached a large number of women in a short period of time using a primary care unsolicited postal survey with no reminders. This led to over 1100 women from four family practices returning family history information, with over 10% identified at increased familial risk. Nevertheless, 84% of invited women did not return a questionnaire. Patients who responded and returned a questionnaire had a similar age distribution compared to non-responders. If a similar 10% of non-responders also have an increased familial risk this would represent a large group of women and a significant unmet clinical need. While some selected response might be expected from those with a relevant family history, on-screen reminders and invitations might increase uptake and identification of risk in practice [38]. This study further underlines the potential to readily use decision support when women do consult with concerns about family history or breast symptoms or have cancer family history collected in routine practice such as on registration with practice or starting oral contraception.

To our knowledge, this is the first study of proactive familial breast cancer risk assessment in women with unrecognised risk in UK primary care practice. The US Family Healthware Impact Trial [13], similarly to the current study, recruited 18% of eligible patients by systematic identification using various recruitment modalities. Other similar primary care studies have primarily recruited women opportunistically when they have expressed concerns about this risk and then given a family history questionnaire to complete prior to their next booked consultation [33]. These approaches result in a higher recruitment rate, but we have shown in our current study that using only opportunistic approaches will lead to fewer women identified who would benefit from preventive interventions and/or surveillance. Like other studies that have used decision support tools in primary care with structured family history collection [38], in this study, a high proportion of women were still classified at increased risk, following specialist assessment (85%).

Following specialist assessment, recommendations for surveillance, genetic testing and chemoprevention were aligned with English national (NICE) guidelines, recent studies and best practice in clinical genetics [14, 24, 25]. The reluctance to recommend chemoprevention has been confirmed by analysis of national primary care prescribing data [41]. The lack of prescribing may be explained by national guidelines only suggesting prescribing of chemoprevention to moderate risk women, whilst recommending chemoprevention in high risk women. In clinical practice the latter is usually only started after genetic mutation confirmed in affected relatives [14].

A significant proportion of women who responded and were confirmed to be at increased risk were from younger age groups. As the UK National Breast Screening programme only starts at 50, these women would not have been detected by this screening programme. However, as mentioned earlier, there is strong evidence that surveillance of these younger women with increased familial risk improves disease outcomes [22, 23]. We recognise women from minority ethnic communities and those with low literacy are underrepresented among those women who engaged. We had anticipated a proactive and more systematic approach to identify familial risk, rather than awaiting consultation with family history concerns or symptoms, may be sufficient to reduce inequalities but this was not demonstrated [42]. More active direct strategies for engagement with these underserved populations need to be developed [43].

This intervention study, with robust experimental design, was completed over a relatively short follow-up period, hence not all the referral outcomes could be verified or followed-up. Related to this, longer term outcomes related to genetic testing and cancer diagnoses could not be ascertained. Due to a small number of participants (n = 10) recruited through the four practices adopting usual opportunistic approach, comparison and statistically analysis between the participants in the proactive practices compared to usual care practices was not feasible. The opportunistic recruitment may be improved by patient-specific reminders in electronic health records [38]. Also, comparative data was not collected for specialist practitioners entering the same dataset but given the extremely high level of correct entry by the primary care administrative staff it is not envisaged that specialists would have entered data any more correctly.

This study suggests the considerable promise of proactively engaging women to have familial cancer risk assessment initiated by postal invitation. This is a relatively low cost strategy and can identify women at increased risk of breast cancer, who would otherwise not access the service, such as, younger women. Moreover, familial risk identification is particularly relevant in women under 50 who are not routinely offered mammographic screening, and who currently present at a later stage of breast cancer (with more years of life to lose).

The study demonstrates that in women aged 30 to 60 years, who respond and complete a validated family history questionnaire, up to 10% could be identified at increased breast cancer risk who may require specialist referral. This would require clear management pathways to manage finite specialist resources. Using current national guidelines, as many as 11% of women’s family histories were considered “uncertain risk” requiring primary care physician to discuss these with a specialist. This could be avoided if more refined primary care decision support were introduced. The use of such decision support software should be actively considered in order to support early detection with the role of better identifying those in need of referral and facilitating pathway improvements. Management pathways between primary care and specialists also needs to reinforce recommendation on chemoprevention for women at moderate and high familial risk of breast cancer.

Parallel qualitative study of women’s and primary care providers’ experiences of this approach is needed and will be reported separately. Further research might explore the use of decision support (compared to none) in primary care in a definitive clinical trial evaluating identification of women at increased familial breast cancer risk through systematic recruitment in both arms. Preceding the trial, there needs to be further intervention development to facilitate recruitment of women from underserved populations [44]. Economic evaluation would help identify cost and benefits of systematic assessment and prevention of cancer outcomes. A longer follow-up time frame would be required to ascertain outcomes related to preventive treatment and clinical management of those at high risk. This includes genetic test results, chemoprevention, surgical prevention, and cancer diagnoses.

## Electronic supplementary material

Below is the link to the electronic supplementary material.

Supplementary file1 (DOCX 23 kb)
