# Supplementary material for: Improving primary care identification of familial breast cancer risk using proactive invitation and decision support
Source: Fam Cancer. 2020 Jun 11;20(1):13–21. doi: 10.1007/s10689-020-00188-z (PMC7870768; doi:10.1007/s10689-020-00188-z)
Supplement: Supplementary file 1 — Supplementary file1 (DOCX 23 kb) [file 10689_2020_188_MOESM1_ESM.docx]

**Table S1.** Demographics of non-respondents

|  |  | Non-respondents | |
| --- | --- | --- | --- |
|  |  | (n = 8953) | |
|  |  | n | (%) |
| Age at time of consent | |  |  |
|  | 30-39 | 2501 | (27.9) |
|  | 40 – 49 | 3320 | (37.1) |
|  | 50 – 59 | 2845 | (31.8) |
|  | 60 and above | 287 | (3.2) |
| Ethnicity | |  |  |
|  | White | 5509 | (61.5) |
|  | Black | 108 | (1.2) |
|  | European of Jewish origin | 0 | (0) |
|  | Other^1^  Missing | 65  3271 | (0.7)  (36.5) |

**Table S2.** Family History Trigger for Referral

| **Reason for Referral** | **Number of Participants** | **Referral Rate^1^** |
| --- | --- | --- |
| Total Referrals in Proactive cohort | 128 | 11.3% |
| *Family History of Breast Cancer* | 68 | 6.0% |
| *Family History of Ovarian Cancer* | 18 | 1.6% |
| *Family History of Breast and Ovarian Cancer* | 32 | 2.8% |
| *Family History of Prostate Cancer* | 1 | 0.1% |
| *Family History of Other Cancers****^2^*** | 7 | 0.6% |
| *Referred before received FaHRAS assessment* ***^3^*** | 2 | 0.2% |
| Referrals Instructed by FaHRAS | 64 | 5.7% |
| Referrals after Discussing with Secondary Care | 64 | 5.7% |
| ***^1^****Denominator based on proactive recruitment (N =1127)*  ***^2^****Cancers include; colorectal, stomach, multiple cancers in proband, pancreatic and young sarcomas*  ***^3^****Patients had family history of breast cancer in 2^nd^ degree relatives* | | |
